# Efficient goal attainment and engagement in a care manager system using unstructured notes

**DOI:** 10.1093/jamiaopen/ooaa001

**Published:** 2020-05-13

**Authors:** Sara Rosenthal, Subhro Das, Pei-Yun Sabrina Hsueh, Ken Barker, Ching-Hua Chen

**Affiliations:** o1 IBM Research, Yorktown Heights, New York, USA; o2 MIT-IBM Watson AI Lab, IBM Research, Cambridge, Massachusetts, USA

**Keywords:** evidence-based healthcare management, patient engagement, supervised machine learning, natural language processing

## Abstract

**Objective:**

To improve efficient goal attainment of patients by analyzing the unstructured text in care manager (CM) notes (CMNs). Our task is to determine whether the goal assigned by the CM can be achieved in a timely manner.

**Materials and Methods:**

Our data consists of CM structured and unstructured records from a private firm in Orlando, FL. The CM data is based on phone interactions between the CM and the patient. A portion of the data has been manually annotated to indicate engagement. We present 2 machine learning classifiers: an *engagement* model and a *goal attainment* model.

**Results:**

We can successfully distinguish automatically between *engagement* and *lack of engagement*. Subsequently, incorporating *engagement* and features from textual information from the unstructured notes significantly improves *goal attainment* classification.

**Discussion:**

Two key challenges in this task were the time-consuming annotation effort for *engagement* classification and the limited amount of data for the more difficult *goal attainment* class (specifically, for people who take a long time to achieve their goals). We successfully explore domain adaptation and transfer learning techniques to improve performance on the under-represented classes. We also explore the value of using features from unstructured notes to improve the model and interpretability.

**Conclusions:**

Unstructured CMNs can be used to improve accuracy of our classification models for predicting patient self-management goal attainment. This work can be used to help identify patients who may require special attention from CMs to improve engagement in self-management.

## INTRODUCTION

Care management is a collaborative, patient-centered approach to managing patient health conditions.^1^ In 2015, the US Centers for Medicare and Medicaid Services began reimbursing non-face-to-face care management and coordination services for certain Medicare beneficiaries with multiple chronic conditions.^2^ Globally, a similar trend has also been pushing the creation and reimbursement of similar health services for better integrated care in countries such as Germany, Ireland, and the United Kingdom.^3–5^

An essential role in care management is that of the care manager (CM), who coordinates with other clinicians, as needed, to set appropriate self-management goals for patients, and who also coaches the patient toward achieving those goals. A CM is usually a licensed nurse, social worker, or other certified specialist. Care management can be delivered to the patient in various settings (eg, at home, in clinic) and via different modalities (eg, telephone, video conference, SMS, chatbot, postal mail, email or other messaging systems). In this work, we focus on the situation where patients have either been recently discharged from the hospital and require transitional care management, or who are self-managing a chronic disease indefinitely. In both situations, the patients in our study are being contacted by a CM on a regular basis, via telephone. We refer the reader to our prior work for further details on the care management model used in our analysis.^6^^,^^7^ Peikes et al.^8^ provide a more general description of care management models used in practice.

One aspect of care management is measuring how often self-management goals are attained. For example, a typical goal for patients who are newly diagnosed with diabetes is to educate themselves about their disease; this includes learning how to recognize disease symptoms and understanding how to perform relevant disease management and medication regimens. Typically, the more engaged a patient is, the easier it is for the CM to coach them toward their goals. The meaning of *patient engagement* is widely discussed in the literature,^9–11^ and not surprisingly, may be defined in a variety of ways to accommodate the different contexts and stakeholders involved. In our work, we consider a patient to be *engaged* if the CM reports having had positive or constructive communications with the patient, or reports that the patient is taking specific actions intended to promote their own health. The objective of our work is to support CM decisions by predicting if a patient is *engaged* or *not engaged* to participate in their own care, and whether they will *efficiently or inefficiently attain an assigned self-care goal*. Henceforth when we refer to our model predicting *engagement* we are referring to both *engagement* and *lack-of-engagement* and predicting *efficient goal attainmen*t as both *efficient* and *inefficient goal attainment*.

CMs typically document each encounter with the patient in electronic care management systems. The CM’s documentation, or records, may include unstructured, natural language notes, as well as more structured entries for the date, time and mode of communication, the patient’s health status, goals and interventions that may have been assigned, and the patient’s progress against those goals. By analyzing both structured and unstructured elements of the CM’s records, we develop models for predicting efficient goal attainment. In particular, we are interested in analyzing CM notes (CMNs) because they often contain cues that indicate the engagement level of a patient to their goals. An example of engagement-bearing language might be “patient taking the medication as prescribed”, while an example of non-engagement-bearing language might be “She says she did not like the way the medication made her feel so she has stopped taking it”. We hypothesize that these cues can help detect the engagement level of a patient and whether efficient goal attainment (and therefore better health outcomes) can be expected.

Furthermore, this work aims to provide support to the CM in the cases where a goal is *not* being obtained efficiently. Providing extra guidance in these cases can help the CM assign new goals that are more likely to result in a positive outcome. Some anecdotal examples are (1) a patient may express that they have been missing follow-up appointments due to lack of transportation can guide the CM to assign the implementation goal or (2) a diabetic admitting to not following their strict diet can indicate that education and self-care goals could be important.

In this article, we focus on answering 2 key questions: First, whether the *engagement* level of a patient can be determined from what is recorded in CMNs; and second, whether the task of *predicting efficient goal attainment* can be further facilitated using the engagement-related signals extracted from CMNs, beyond what has been captured from the structured entries of CM transaction records. Our contributions are:

- We identify an opportunity to leverage unstructured data (text) from CMNs to improve learning models to assist in care management.
- We combine structured and unstructured features to develop models for predicting efficient and inefficient patient goal attainment.
- We build on prior work to create an engagement model with significant improvements in performance using domain adaptation techniques and BERT (Bidirectional Encoder Representations from Transformers)^12^ to generate cues and unstructured features for the *efficient goal attainment* task.

## RELATED WORK

We focus on 2 areas of related work: learning patient engagement from unstructured notes, and patient engagement and goal attainment for care management.

### Learning patient engagement cues from unstructured notes

Previous studies have demonstrated the application of natural language processing (NLP) techniques for capturing relevant information from clinical notes taken during clinical visits (including both physician and nurse notes, as part of the patient’s clinical records). Examples include: (1) identifying patients with specific diseases^13^; (2) monitoring adverse events as a patient safety surveillance tool^14^; (3) supporting clinical decisions to manage risk around chronic conditions^15^; (4) predicting readmission and phenotyping from discharge summaries^16^; (5) extracting health quality information (eg, identifying care gaps)^17^; (6) extracting problem lists to improve clinical work productivity^18^; (7) assessing doctor compliance, sentiment, and adherence (eg, compliance to tobacco cessation guidelines)^19^; and (8) protocol compliance in discharge summaries.^20^ Among these, only a few investigate linking information from notes with structured records. For example, Watson et al.^21^ link the social health determinants identified from both structured records and clinical notes to predict re-admission.

CM records, on the other hand, capture information about a patient between these clinical visits and hospital stays and can provide insight into the self-management activities of the patient in the home (ie, non-clinical) setting. Lopez et al^22^ Explore consolidating and highlighting relevant information from multiple care management and clinical notes but they do not specifically address engagement. Other work in care management data analytics has been used to identify higher priority patient cohorts (eg, high-cost, high-need patients)^23^ and to surface social determinants of health along with clinically relevant information at the population (or cohort) level.^24^ These applications of CM data analytics have not fully explored the potential of using care management records (especially CMNs) to enhance CM work productivity and increase patient understanding at the level of the individual. Previous studies show that using informatics tools to personalize health communication with patients is important to patient engagement.^25^^,^^26^ Evidence has also shown that patients who are more engaged have better health outcomes and care experiences.^27^

While there exists work dealing with sentiment in clinical notes, such as positive or negative affect,^28^ and speculative language,^29^ lack of engagement cannot be reduced to sentiment. For example, lack-of-engagement bearing language can also contain positive sentiment: “patient is feeling better so she has stopped taking her medication”. Topaz et al.^15^ developed a document level discharge note classification model that identifies the adherence of a patient in the discharge note. However, they focus only on the lack of adherence, specifically, toward medication, diet, exercise, and medical appointments. In contrast, we follow prior work and also examine the quality of the patient’s communication and relationship with the CM, which may be another indication of engagement.^30^

### Patient engagement and goal attainment for care management

Previous work has shown that goal attainment is important to the implementation of health behavior change and can serve as a proximal proxy measure of patient health outcome in the longer term.^31–33^ In the domain of care management, previous work has focused on learning individual-level care planning strategies from practice by the Behavioral Response Inference Framework,^6^^,^^7^ which outperformed population-level strategies in the task of delivering more accurate intervention recommendations for goal attainment. In other domains, such as social media analytics, goal attainment records have been constructed from social media messages using NLP techniques to understand action–outcome relationships and the propensity of a user for taking actions to achieve health outcomes.^34^^,^^35^ However, to date, no other studies have investigated how to leverage NLP approaches to predict the attainment of health goals, where these goals are primarily attained through the active engagement of the patients themselves.

In our work, we aim to bridge the gap by first understanding how to extract engagement cues and quantify engagement level from unstructured CMNs. Then, we combine the extracted signals with other goal attainment-related features from care management records to determine what the potential is for efficient goal attainment under current engagement and intervention strategies.

## DATA

Our first dataset (referred to as the GOAL dataset) was curated from a private, not-for-profit healthcare network in the southeastern United States over a 24-month period from 2015 to 2017. It contains records of patients recently assigned to a disease management program or a transitional care program after being discharged from the hospital (disease management programs focus on addressing chronic care; transitional care programs aim to reduce hospital re-admissions). The CM records contain goal attainment transactions across 30 different goals in 6 major focus areas. The entire dataset consists of 4504 transition of care and 440 chronic care patient interactions. In order to ensure a good sample of language use in engagement and goal attainment, we restricted the analyses to those records that contain at least one non-empty CMN, giving 2710 goal attainment records from 1882 unique patients’ care plans. Repeated encounters for a particular patient for a particular goal were aggregated together into a single note. The GOAL dataset consists of 2 parts: (1) structured CM transaction records and (2) unstructured CMNs. From the 2710 records, we obtained goal attainment history from the structured entries of the records. A total of 2363 goals were attained efficiently (ie, in less than or equal to 2 weeks). Three hundred and forty-seven goals either were not met or took more than 2 weeks to complete, with some taking more than 180 days to complete.

### Structured CM transaction records

**Figure 1.**
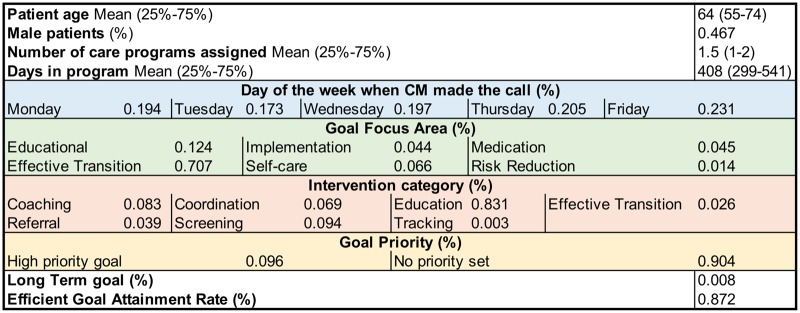
Summary statistics of the GOAL dataset. Efficient goal attainment is defined as achieving the assigned goal in less than 2 weeks.

The CM records include structured information gathered by the CM in the following 4 categories for each patient: (1) patient-specific information (eg, age, gender); (2) CM program experience (eg, how many care programs have been assigned to the individual, what type of care program); (3) call-specific (eg, on which day of the week was the call made); and (4) goal and intervention assignment (eg, what type of goal—focus area, short-term vs. long-term—and whether it is a priority). Note that any given goal can have multiple interventions being assigned for its attainment. A summary of the GOAL dataset statistics for each of the structured data categories is shown in Figure 1.

### Unstructured CMNs

In addition to recording structured information, CMs record notes based on phone calls with the patient. To answer the 2 main questions in this study, we follow the notion of patient engagement from CMNs described in previous work^30^ but take a different approach in our model and setup. Although we cannot include an actual note, we provide some example sentences in Table 1.

**Table 1.** Descriptions and anecdotal examples for engagement and lack of engagement

Label	Description	Examples
Engagement with care	The patient is engaged in their well-being by describing/exhibiting healthy behavior, positive outlook, and social ties.	“Patient disappointed by lack of weight loss but is just beginning exercise regimen”; “Patient joined book club.”
Engagement with care manager	Adherence to a doctor or CM instruction or understanding of CM advice.	“Patient verbalized understanding”; “Patient confided that she has gaps in nitroglycerin use.”
Lack of engagement with care	Lack of engagement by using language suggestive of non-adherence to guidelines, health-adverse behavior, lack of social ties, or negative impression of patient self-care.	“White female, disheveled appearance”; “Patient admits to ‘sedentary’ lifestyle.”
Lack of engagement with care manager	Non-adherence to a prescribed instruction or a negative response to interaction.	“Patient rude during call”; “Patient angrily refused further outreach.”

Recent trends in medicine have focused on the role of patient behavior and lifestyle choices in positive health outcomes.^36^^,^^37^ As described in prior work,^30^ specific behavioral characteristics of patient engagement that we identified in the notes included whether the patient was reported to have had constructive communications with the CM (ie, engaged with CM) and whether the patient has taken specific actions to promote their own health (ie, engaged with care); we annotated the GOAL dataset accordingly for engagement or lack-of-engagement in the CMNs on the sentence level. A description of the data labels is shown in Table 1. (Although the labels in the dataset distinguish between patient engagement with care and patient engagement with the CM, we do not make a distinction in the target of the engagement in our classifier.^30^) These annotations were done in-house with detailed feedback from practicing CMs and experts in care management solutions. In the annotation process, multiple rounds of cross-evaluations were employed to ensure the consistency of labeling. The amount of data that was labeled as engagement (409 sentences) or lack-of-engagement (331 sentences) is relatively small with 740 sentences in total. This is because most sentences do not indicate patient engagement. While Rosenthal and Faulkner^30^ included the class “other”, we chose not to include this class because its frequency dominates the model during training. Instead, we trained and tested the model only on data with engagement or lack-of-engagement labels. To address the small data size, we obtained a large amount of engagement text freely from tweets in a health-related Twitter dataset.^38^ We automatically labeled 128k tweets from the Twitter dataset with engagement (118k) and lack-of-engagement (10k) by looking for words such as “take” and “keep” for engagement and “refuse” and “deny” for lack-of-engagement.

### Method

To answer the 2 motivating questions, we have defined 2 tasks for which we train models: (1) *Engagement classification* based on unstructured CMNs; (2) *Efficient goal attainment classification* based on both engagement features from unstructured CMNs and structural features from CM transaction records.

### Engagement classification

Our first task is to classify the sentences in CMNs as signaling engagement or non-engagement behavior using the GOAL and Twitter training datasets described in the Data section. The test dataset contains data from the GOAL dataset only.

We experimented with classifying engagement using 3 different models. The first is a support vector machine (SVM) model—a traditional feature-heavy machine learning approach used in prior work.^30^ We experimented with the same 6 groups of features: *n-grams and parts-of-speech*, *embeddings*, *lexico-syntactic* (words and dependency tuples), *lexical-stat* (word statistics, such as sentence length and word lengths), *sentiment*, and *medical* (identifiable medical concepts). We used the python scikit-learn SVM linear model.^39^ As in the prior work, we explored domain adaptation, in this case using the health-related Twitter dataset as the out-of-domain data.^38^ Following the approach of Daumé,^40^ we used domain adaptation with each feature duplicated for the target and source languages, with the feature only turned “on” for the target language. One major difference between our experiments and those reported in prior work is that we excluded the “other” class during training. Our second approach uses deep learning, specifically, a recurrent neural network (RNN) model with attention implemented in TensorFlow.^41^^,^^42^ In our model, we used attention to generate a weighted sum of GRU cell outputs for each word in the input text, to focus the model on those words that are the most useful for prediction. We used the Adam optimizer, a batch size of 128, dropout of 0.2, learning rate of 0.001, and an embedding size 300 for 200 epochs. Finally, several recent studies have shown that using the pre-trained language model BERT^12^ significantly improves performance of down-stream tasks. The BERT model is the state-of-the-art language representation model that pre-trains bidirectional representations by jointly conditioning on both left and right context. In our setting, we fine-tune the BERT model on our engagement training data and take the output for the first token in the input (ie, the special [CLS] word embedding) as the representation of the input. It is then used for label prediction by feeding into a classification layer. For our BERT experiments, we used a PyTorch implementation (https://github.com/huggingface/pytorch-pretrained-BERT) with the bert-base-uncased model. We used the default BERT parameters including the BERT Adam optimizer, a batch size of 32, dropout of 0.1, and embedding size 768. All text was cutoff to the first 128 word-pieces and we ran it for 3 epochs.

### Efficient goal attainment classification

Prior work focused on general goal attainment which was defined by a structured field indicating that the goal was met or not met.^25^^,^^26^ The problem with using this field is that a goal could take 6 months to complete. While the goal might have been attained eventually, it might not be the best choice to recommend to the target patient. Therefore, our second task is to predict whether a goal specified in a CM record can be achieved efficiently. We define efficiency as a goal being completed in less than or equal to 2 weeks, which is the usual period of time CMs are instructed to spend attempting to encourage individual patients to achieve a particular goal. Even though we define this task as efficient goal attainment, being able to predict the goals that will not be completed efficiently is just as important (if not more so), because the CM should focus on these patients. Predictions of inefficient goal attainment would be surfaced to the CMs when they reach out to the patients, so as to facilitate shared decision making on how to intervene for achieving an assigned health goal.

We first processed the structured call records data and fed it into our pipeline to identify the differentiating factors for goal attainment. The key components of the pipeline are: (1) extract the behavioral features in an online dynamic fashion from the call records, (2) extract the engagement features and n-grams from the CMNs, and (3) perform an outcome-driven feature projection using locally supervised distance metric learning.^43^^,^^44^

We generated 4 groups of features:

*struct*: 25 structured features (Since we are predicting the efficiency of goal attainment in this task, we did not include any structured data that overlaps with efficient goal attainment (eg, whether an assigned health goal has passed its due date is directly correlated to the structured field used to compute efficient goal attainment.) such as gender, age, and goal focus areas. All features are shown in Figure 1.

*eng*: The best engagement model (BERT) was used to generate 8 engagement features. We generated the max, count, median, and mean scores for both engagement and lack of engagement per note by running the model on all of the train and test sentences in the notes.

*eng_n-gram*: In addition to the *eng* features we also retained the sentences from the engagement model that were representative of engagement and lack-of-engagement to explore important words from each class.

*n-grams*: The most frequent words and pairs of words from the unstructured notes.

**Figure 2.**
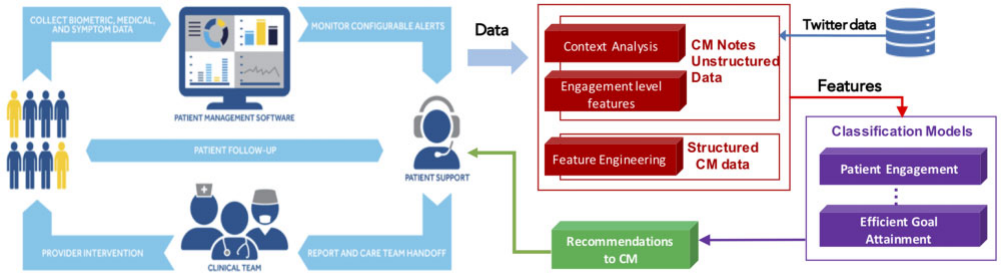
The pipeline of the entire system. The left portion illustrates the typical role of the care manager in the patients care. The right portion illustrates how our system aids the care manager.

The combined structured and engagement features make up our patient representation for efficient goal attainment outcome and allow for an interpretable model that can provide the CMs with meaningful insight for assigning goals to patients. We used these features in a linear SVM model using scikit-learn.^39^ The pipeline of the entire system is shown in Figure 2.

## RESULTS

In this section, we describe in detail the results for our 2 models: engagement classification and efficient goal attainment classification.

### Engagement classification

The engagement results for each model are shown in Table 2. Experiments were performed on a held-out test set of approximately 20% of the data. We explored different permutations of features for the SVM model, but using all of the features worked best so we include only these results in the table. Exploiting out-of-domain Twitter data with the SVM shows significant improvement, from 85% to 89% average *F*-score. On the other hand, applying transfer learning using Twitter data with the RNN does not provide any improvement. Although the RNN model does perform slightly better than the SVM model with just CMNs, the SVM model trained with Twitter data outperforms the RNN model. We expect this is due to the very small size of the in-domain data. Deep learning approaches tend to perform better when a large amount of data is available. Finally, using the BERT model fine-tuned on the CMNs gives us significantly better results compared to both the SVM and RNN, with an average *F*-score of 95%.

**Table 2.** Precision, recall, and *F*-score results for engagement prediction using SVM, RNN, and BERT models

		Engagement			Lack of engagement			Average		
Model	Datasets	*P*	*R*	*F*	*P*	*R*	*F*	*P*	*R*	*F*
SVM	CM	0.80	0.93	0.86	0.91	0.75	0.83	0.85	0.85	0.85
SVM	CM + Twitter	0.83	0.98	0.90	0.98	0.79	0.87	0.89	0.89	0.89
RNN	CM	0.83	0.90	0.87	0.88	0.81	0.84	0.86	0.86	0.86
RNN	CM + Twitter	0.82	0.92	0.87	0.90	0.79	0.84	0.86	0.86	0.86
*BERT*	CM	0.95	0.95	0.95	0.95	0.95	0.95	0.95	0.95	0.95

*Note*: Significantly outperforms all other models (SVM CM + Twitter *P*-value ≤ 0.1 and all other models *P-*value ≤ 0.01.)

### Efficient goal attainment classification

We trained an SVM model using different combinations of features as the training feature sets and used the development feature set to tune the model parameters. We ran experiments with 2 baselines and the 4 feature groups described in the previous section: *struct*, *eng*, *n-grams*, and *eng-n grams.*

We compare our model against a majority and minority baseline:

*majority baseline*: predict that all goals are attained efficiently (≤*2 weeks*).

*minority baseline*: predict that all goals are not attained efficiently.

Due to the small size of our dataset and class imbalance we chose to perform 5-fold cross-validation and report the average results over the 5 folds. Although we report accuracy, this is not an ideal measure in this case because we want to find the goals that cannot be met efficiently. Therefore, we also report precision, recall, and *F*-score. We show performance on each class, efficient goal attainment (≤*2 weeks*) and inefficient goal attainment (>*2 weeks*), as well as the macro-average of the 2 classes. Although we provide the results for each class, the accurate picture of our systems’ performance is the average precision, recall, and *F*-score. This stems from the need not only to predict goals that are met efficiently (which could be done with high accuracy by predicting the majority class), but also to find goals that cannot be met efficiently. Statistical significance was computed using McNemar’s test.

In Table 3, we show various combinations of the feature groups and also compare to the baselines. It is important to note that the ≤2 weeks baseline does very well because of the class imbalance. However, this baseline does not predict any of the >2 weeks instances correctly (our aim is to find these rare cases of inefficient goal attainment). In all cases, we significantly beat the majority baseline in average *F*-score. Furthermore, the 3 models that combine struct and engagement features significantly beat the model using just the struct features. Our best result is to include the structured, engagement, and n-gram features. This combination gives an average *F*-score of 0.66. These results indicate that combining engagement with the structured features does help and that the unstructured notes provide important cues to engagement and efficient goal attainment. It is also worth noting that the classifier *n_grams*, which uses only n-grams as features (without any structured features) performs very comparably to the classifier *struct*, which uses only structured features. This indicates that the n-grams are informative on their own.

**Table 3.** The 5-fold cross-validation results for efficient goal attainment prediction in terms of accuracy, precision, recall, and *F*-score for the structural (*struct*), engagement (*eng*), n-grams, and engagement-oriented n-grams (*eng_n-grams*) groups of features

	Average				≤2 weeks				>2 weeks			
	*A*	*P*	*R*	*F*	*A*	*P*	*R*	*F*	*A*	*P*	*R*	*F*
Majority (all efficient)	**0.87**	0.44	0.50	0.47	**0.87**	0.87	1.00	**0.93**	**0.87**	0.00	0.00	0.00
minority (all inefficient)	0.13	0.06	0.50	0.11	0.13	0.00	0.00	0.00	0.13	0.13	**1.00**	0.23
struct	0.72	0.63	**0.77**	0.63	0.72	**0.97**	0.71	0.82	0.72	0.29	0.83	0.43
struct + eng	0.74	0.63	**0.77**	0.64	0.74	0.96	0.73	0.83	0.74	0.30	0.81	0.44
struct + eng + n-grams	0.77	**0.65**	**0.77**	**0.66**	0.77	0.96	0.77	0.85	0.77	**0.33**	0.78	**0.46**
struct + eng + eng_n-grams	0.76	0.64	**0.77**	0.65	0.76	0.96	0.76	0.85	0.76	**0.33**	0.79	**0.46**
n-grams	0.72	0.62	0.75	0.62	0.72	0.96	0.71	0.81	0.72	0.28	0.79	0.42
eng_n-grams	0.64	0.59	0.70	0.55	0.64	0.95	0.62	0.75	0.64	0.23	0.77	0.36

*Note*: All models significantly beat the *Average F-score* for the baselines and the *underlined* models significantly beat the *Average F-score* of the *struct* model with *P*-value ≤ 0.001. The boldface values indicate the highest score in each column.

Looking at the top structured features that the SVM correlated with the prediction for both classes in the baseline model for all the folds, we found that the focus areas *educational*, *self-care*, *reducing risks*, and *medications* were always correlated with efficient goal attainment, whereas *other* and *implementation* were always correlated with inefficient goal attainment. Goals that were marked as *high priority* and *long term* were also indicative of efficient goal attainment. On the other hand, *number of care programs assigned* and having no *priority* set were indicative of inefficient goal attainment. Both of these can indicate a lack of response from the patient.

We find that general n-grams performed better than including n-grams based on the engagement model. We expect this is because there are overlapping words in both classes. The SVM model learns how to distinguish between the words regardless of the prediction for the sentence. To show these observations, we looked at the top 10 features per class of our best model (*struct* *+* *eng* *+* *n-grams*) for each of the folds to see which n-grams (words or bi-grams) occurred most frequently in each class. These n-grams are shown in Table 4. Many of the top n-grams in the ≤*2 weeks* class involve successfully performing the “welcome home call” (eg, “wh spoke”) indicating a responsive patient. Many of the top n-grams in the >*2 weeks* class are connected to the patient saying or admitting something (eg, “patient admitted”). These can indicate that the patient admitted that they were not complying or were not interested in their goals. These words can be cues and provide insight to the CMs as to whether the assigned goal and intervention were successful for the patient.

**Table 4.** The top n-grams for each class sorted by frequency of occurrence in the cross-validation folds

	≤2 weeks	# folds	>2 weeks	# folds
1	wh (*welcome home*) spoke	4	Home patient	5
2	Needs arise	4	pt (*patient*) states	4
3	Welcome	4	hh (*home healthcare*)	4
4	dm (*diabetes management*)	4	Discharge	3
5	Left	3	didn	3
6	Did questions	2	Patient admitted	3
7	Completed	2	Fever	2
8	Needed	1	Medications	2
9	Health	1	Informed	2
10	Therapy	1	pt (*patient*) said	1
11	2017	1	Discharged	1
12	Questions concerns	1	Arise	1
13	Symptoms	1	Denies	1

*Note*: The acronym expansions provided in parenthesis are shown for clarity but do not appear in the notes.

## CONCLUSION

In this article, we hypothesized that we could predict efficient goal attainment with greater performance than a proposed baseline model. Furthermore, we explored using engagement features derived from unstructured CMNs for improving the prediction of efficient goal attainment, compared to using only features derived from structured care management records. To test our hypothesis, we trained models for patient engagement classification and models for efficient goal attainment classification.

Comparing the *engagement* models we implemented showed that the BERT model outperformed models that were pre-trained on an external cheaply labeled data set (RNN and SVM). We note that the performance of the latter models were still very reasonable (ie, *F*-scores > 0.8). We conclude that transfer learning approaches are worth exploring in this setting.

Results of our *efficient goal attainment* experiments showed that the (*F*-score) performance of our prediction models outperformed the majority baseline model by around 40%. This performance improvement can be attributed to the ability of our models to improve the precision associated with detecting inefficient goal attainment. Predicting inefficient goal attainment is challenging in this dataset because these events constitute a minority of the events in the data. In practice, however, identifying these instances could have an impact on the practice of care management, by helping CMs to identify patients that are at greater risk for not achieving their goals. These patients may benefit from more extensive assessment and interaction with CMs. Our findings show that the improvement when incorporating engagement features from the unstructured notes is statistically significant compared to models using features from structured data alone. Second, we found that our *n_grams* model performed comparably to our *struct* model, and considerably better than our *eng_n_grams* model for predicting goal-attainment. Since the *n_grams* model was not restricted to sentences indicating engagement, our finding is consistent the findings of Lopez et al.,^21^ which learn non-engagement-related patient attributes from care notes.

Aside from being useful for predicting efficient goal attainment, the classification of engagement is independently useful for surfacing specific engagement strengths/weaknesses of the patient that could be addressed. In particular, the aspects of engagement that our models can detect would be useful for highlighting potential issues in the quality of the communications between the patient and the CM, or specific health behaviors that could be further encouraged or discouraged. Inspection of the cues in unstructured text may even provide insight to the CMs that are suggestive of the types of goals and interventions that could be most appropriate. In the future, we intend to expand our analysis by clustering patients to discover the types of goals and interventions that may be most appropriate for patients from each cluster.

## AUTHOR CONTRIBUTIONS

SR, SD, P-YSH, KB, and C-HC were involved in study design and data preparation/annotation. SR implemented the NLP-based engagement classification, domain adaptation, and transfer learning models. SD and P-YSH were involved in structured data analysis and feature engineering. SD, SR, and P-YSH implemented the code for efficient goal attainment. All authors were involved in interpretation of the results and drafting/revising the manuscript. All authors have approved the final manuscript.
